# Follow-up study on lead exposure in children living in a smelter community in northern Mexico

**DOI:** 10.1186/1476-069X-10-66

**Published:** 2011-07-18

**Authors:** Marisela Rubio-Andrade, Francisco Valdés-Pérezgasga, J Alonso, Jorge L Rosado, Mariano E Cebrián, Gonzalo G García-Vargas

**Affiliations:** 1 Facultad de Medicina, Universidad Juárez del Estado de Durango, Gómez Palacio, Durango, México; 2 Escuela de Ciencias Naturales, Universidad Autónoma de Querétaro, Querétaro, México; 3 Sección Externa de Toxicología, Centro de Investigación y de Estudios Avanzados del IPN, Mexico, DF, México; 4 Instituto Tecnológico Regional de la Laguna, Torreón, Coahuila, México; 5 Biosensor Group, Universidad Autónoma de Barcelona, España

## Abstract

**Background:**

To study the changes of children lead exposure in the city of Torreon during the last five years, after environmental and public health interventions, using the timeline of lead in blood concentration as the biomarker of exposure and its relation to lead in soil concentrations.

**Methods:**

This follow-up study started in 2001 and consisted of 232 children living in nine neighborhoods in Torreon. Children were tested at 0, 6, 12 and 60 months. Lead in blood concentrations, Hemoglobin, Zinc-Protoporphyrin, anthropometric measures and socioeconomic status questionnaire was supplied to the parents.

**Results:**

Median and range of lead in blood concentrations obtained at 0, 6, 12, 60 months were: 10.12 μg/dl (1.9 - 43.8), 8.75 μg/dl (1.85 - 41.45), 8.4 μg/dl (1.7 - 35.8) and 4.4 μg/dl (1.3 - 30.3), respectively. The decrease of lead in blood levels was significantly related to ages 0, 6, 12 and 60 months of the follow-up study. The timeline of B-Pb was associated with the timeline of lead in soil concentrations.

**Conclusions:**

B-Pb levels have significantly decreased in the group of children studied. This could be explained by a) environmental interventions by authorities and the smelter companies, b) normal changes in hygienic habits as children age and c) lead redistribution from blood to hard tissues.

## Background

The growth of industry in northern Mexico has created the potential for severe environmental problems. Yet, attention to environmental controls has lagged behind the pace of industrialization. Industrial sites such as ore smelters are often located in residential areas, where exposure to heavy metals through inhalation and ingestion of contaminated soil and dust is possible [1].

Lead has long been established as a health threat to people living in communities surrounding ore smelters and refineries [2]. In 1999, the Benin group [1], analyzed dust samples obtained from residential neighborhoods around metallurgic industries in Monterrey, Chihuahua and Torreon, Mexico. Dust concentrations of all heavy metals were significantly higher around the active smelter in Torreon, where more than 90% of samples exceeded Superfund cleanup goals of 400 μg/g for lead in soil [3]. The dust samples in Torreon had a median lead concentration of 2,448 μg/g [interquartile range (IQR): 179-4,884 μg/g] with a 95th percentile of 13,231 μg/g. Torreon Coahuila, the largest city (> 520,000 inhabitants) of the Lagunera region, is located in central northern Mexico. It hosts the largest nonferrous metallurgical complex in Latin America, which is also the fourth largest in the world. This smelter was built in early 1900 and processes approximately one third of the lead ore in Latin America [4]. In 2010, it produced 142,200 metric tons of lead, 232,700 metric tons of zinc, 103.3 million ounces of silver and 1,093 million ounces of gold [5].

The impact of the smelter complex on environmental health has been recorded since 1977 [6], when it was reported that persons from Torreon had the highest levels of lead in hair when compared to other cities in the country. Early ecological works from 1982, also reported high levels of B-Pb in children in Torreon [7]. Between 1997 and 2001, we published the results of several studies on lead environmental contamination in Torreon [8,9]. The main findings were that heavy metal contamination in soil and dust around the smelter site far exceeded background levels. We also reported that a high proportion of children (> 95%) living in the area had lead in blood (B-Pb) levels greater than 10.0 μg/dl [B-Pb level in children that merits intervention according to the US Centers for Disease Control and Prevention (USCDC), conversion factor for blood lead concentrations (μg/dl) × 0.04826 = (μmol/L)] [10]. Lead concentrations in soil and B-Pb were inversely related to the distance from the lead smelter. This data supported the fact that the smelter complex was and continued to be the main source of lead emission in the city.

Ingestion appeared to be the main exposure mechanism for lead entry to the organism after soil and dust. Soil was the major contaminated environmental media, having concentrations above 400 μg/g, the hazard standard level for children's playground areas in residential soil and above 1,200 μg/g in the neighborhoods, which is the hazard standard level in bare soil in non playground residential areas [11]. In our previous study [8], we estimated the magnitude of lead exposed children in 1999, according to the recommended action categories by the US CDC [10], the population aged 0 to 14 years living in the area and officially recognized to be contaminated with lead, was calculated above 14,950 individuals^1^. Based on this data, we concluded that approximately between 12,000 and 13,500 children had B-Pb levels above 10 μg/dl. It was also estimated that between 1,250 and 1,440 children had B-Pb levels greater than 45 μg/dl and were in need of environmental, medical and possibly, pharmacological interventions; these data of the Ministry of Health screening confirmed the proportion of lead poisoned children described in our study. Furthermore, in the same report, twenty-six children (0.44%) were identified as having B-Pb above 70 μg/dl.

During 1999, the Mexican Health and Environmental authorities in conjunction with the smelter company and under the surveillance of nongovernmental organizations started a vast program based on an official decree from the Government of the State of Coahuila and several official regulatory agencies of the Mexican Federal Government^2^. The program consisted of technological and engineering changes in smelter complex practices to decrease metal emissions and several cleaning and remediation activities. Among them, the most important was the collection of more than 100,000 Kg of dust containing high concentrations of metals in streets, highways, roofs and houses using high efficiency vacuum machines; this action was important as an environmental intervention house by house. Additionally, more than 100 families from the neighborhood with the highest levels of lead pollution were relocated to sites far away from the smelter. The program was carried out within a 3 km radius of the center to the smelter complex. In parallel, the State Ministry of Health opened a clinic with toxicological, pediatric, allergology, neurology, psychology, nutritional interventions, social work and nursing services.

In this study, we evaluated the changes of lead exposure in Torreon during the five years since the launch of the environmental and public health interventions, we studied the timeline of lead in blood concentration, which is the main biomarker of exposure for lead in the group of children studied and its relation to lead in soil concentrations.

## Methods

### Subjects and design of the study

**Figure 1:**
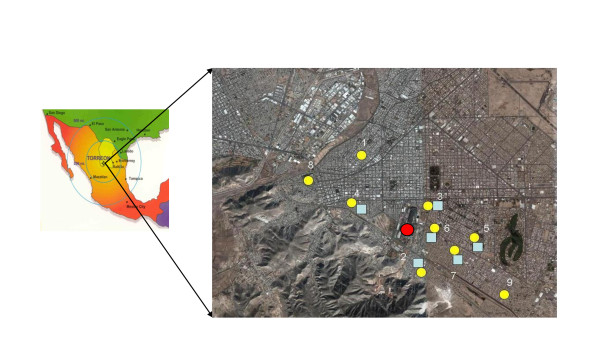
**Geographic locations of each of the nine elementary schools and the smelter complex in the city of Torreon, State of Coahuila, Mexico**. Yellow circles represent school locations and blue squares represent soil sampling sites. Red circle represents the site of the Smelter Complex in Torreon City.

This study, which began in January 2001, consisted of children living in nine neighborhoods in Torreon. Children were recruited at 6-8 years of age, at a time they start elementary school [12]. Children qualified for the follow-up study if they attended the first grade in one of the nine public elementary schools located within 3.5 km of a metallurgic smelter complex in Torreon (Figure 1). Exclusion criteria include severe diseases, antecedent of chelating treatment for lead poisoning, B-Pb concentrations > 45 μg/dl, and pregnancy. Children were tested at 0, 6, 12 and 60 months. The number of children participating was 598, 517, 481 and 232, respectively. The main reasons given by non participating families (except in four children, in most cases, there were a family per participating child) were: a) they moved or migrated and it was not possible to locate them in the same neighborhood (85.6%); b) they declined to participate (10.2%); c) repeatedly missed appointments (3.87%) and d) two children died during the five years of follow-up due to leukemia in the first case and a complicated appendicitis in the second case (0.33%). In Table 1 we show the number of children that remains in each phase of the follow-up. The parents of the studied children gave informed written consent before being enrolled. This study was approved by the Institutional Review Board of Juarez University of Durango State (IRB00004485; FWA00012172).

**Table 1:** Timeline of B-Pb (μg/dl), in lead exposed children during 60 months of follow-up by school.

		Time of Follow-up (months).			
School number.		0	6	12	60

1	n	79	65	59	29

	Median	7.52	6.95	6.10	3.68

	Minimum	1.99	1.86	1.7	2.3

	Maximum	34.62	27.0	25.60	10.4

2	n	71	57	51	34

	Median	13.94	13.20	12.20	4.75

	Minimum	5.38	5.35	4.70	2.02

	Maximum	43.80	41.45	35.80	30.30

3	n	97	83	73	15

	Median	9.99	8.70	9.30	4.53

	Minimum	3.90	3.85	3.95	1.78

	Maximum	41.93	40.6	34.35	7.2

4	n	51	45	43	18

	Median	12.55	10.40	9.55	5.48

	Minimum	5.83	4.05	4.15	3.48

	Maximum	34.72	26.10	25.90	12.58

5	n	58	51	50	32

	Median	8.68	6.40	7.30	5.49

	Minimum	3.0	2.90	3.10	3.36

	Maximum	20.05	16.60	18.45	8.7

6	n	70	58	55	14

	Median	14.20	11.10	12.55	3.27

	Minimum	3.30	3.80	3.30	1.30

	Maximum	41.50	36.75	35.80	9.13

7	n	52	51	47	27

	Median	10.70	8.55	8.20	4.08

	Minimum	4.40	2.90	4.25	2.35

	Maximum	22.20	18.15	17.65	7.44

8	n	72	66	66	40

	Median	9.10	8.68	8.18	3.30

	Minimum	3.70	3.45	4.05	1.43

	Maximum	17.30	16.0	13.95	7.67

9	n	48	41	37	23

	Median	7.25	5.85	5.80	4.58

	Minimum	2.30	2.50	2.40	2.07

	Maximum	16.90	14.90	16.45	12.70

Total	n	598	517	481	232

	Median	10.12	8.75	8.30	4.40

	Minimum	1.9	1.85	1.7	1.30

	Maximum	43.8	41.45	35.8	30.3

Median, minimum and maximum are expressed as B-Pb concentrations expressed in μg/dl units.

### Lead in blood determination

A venous blood sample was collected from each child after an overnight fast. Blood was collected in 5 mL EDTA-vacutainer trace-metal free tubes (Becton Dickinson, Franklin Lakes, NJ). Samples were transported in a cooler to be processed in the laboratory on the same day. For B-Pb measurements, samples were analyzed in duplicate using Atomic Absorption Spectrophotometry (Zeeman-Graphite Furnace-Analyst 800, Perkin Elmer Norwalk, CT), according to Miller et al. [13] and those with a CV > 5% were re-analyzed. In human Toxicology, lead in blood concentration is usually expressed in μg Pb/dl of blood, for conversion to SI units we can use the conversion factor for SI units: 1 μg/dl = 0.04826 μmol/L. Lead in bovine blood (SRM 955b, National Institute of Standards and Technology) was used as the standard reference. The laboratory participates in quality control programs were the Inter Laboratory Program of Quality Control at Zaragoza, Spain, and the Wisconsin State Laboratory of Hygiene Program (WSLH).

### Other biochemical analyses

Hemoglobin (Hb) was analyzed using a Hemocue Photometer (Hemocue AB). Zinc protoporphyrin (ZPP) was tested in whole blood at the school with ZP Hematofluorometer (AVIV Biomedical).

### Lead in soil determinations

Parallel to this study, other group [14] worked on lead in soil concentrations and their impact on risk assessment of adverse health effects in children. From this effort, we obtained the lead in soil concentrations in the neighboring areas of all the studied schools except those marked with the numbers 1, 8 and 9 (Figure 1). In this study, samples were only taken in a perimeter with radio of 2.5 km from the smelter, because is the area officially recognized as environmentally affected by metal emissions.

In order to compare the lead in soil concentrations to that before environmental intervention began, we presented lead in soil concentrations from 1999 in the same vicinities, quoted in the official report of environmental authorities^2^.

### Statistical methods

Statistical analysis was performed with Stata 8 (STATA Corp., College Station, TX). All results were subject to a general descriptive analysis. B-Pb values presented log normal distributions and were log transformed to fit normal distribution. Pearson correlations and ANOVA between groups of demographic variables were performed to evaluate their association or differences and when needed, they were evaluated with linear regression models.

## Results

### Population data

We were able to locate and accept 232 children to participate in the last phase of the study after 60 months from the initial cross sectional study. Gender distribution had slightly more males but there were no statistical differences between girls and boys (Table 2). The nutritional status study, based on body mass index (BMI), showed that overweight prevalence was consistently higher than underweight prevalence, although these differences proved less significant as the children aged. Interestingly, underweight (BMI < 1 SD) consistently rose from 8.1% at the beginning of the study to 15.3% in pubertal ages.

**Table 2:** General characterization of children studied 60 months in Torreon, Mexico.

	Time of Follow-up (months)			
Variable	0	6	12	60

n	598	517	481	232

Female (%)	47.6	46.5	46.4	47.6

Age (yr)^a^	7.2 ± 0.33	7.7 ± 0.39	8.2 ± 0.37	12.2 ± 0.34

weight (Kg)^a^	24.6 ± 5.2	26.8 ± 5.8	28.8 ± 6.5	48.1 ± 12.1

Height (cm)^a^	120.7 ± 5.3	123.8 ± 5.5	126.9 ± 5.6	151.9 ± 7.0

BMI*^b^	16.2 (13.0 - 29.2)	16.8 (13.0 - 30.5)	17.1 (13.2 - 30.3)	20.6 (12.2 - 35.8)

BMI % < 1 SD	8.1	8.8	12.2	15.3

BMI % > 1 SD	17.7	11.5	14.8	16.2

^a ^*Values are means ± SD.*

^b ^Values are medians (range).

* Body Mass Index.

### Lead exposure

**Figure 2:**
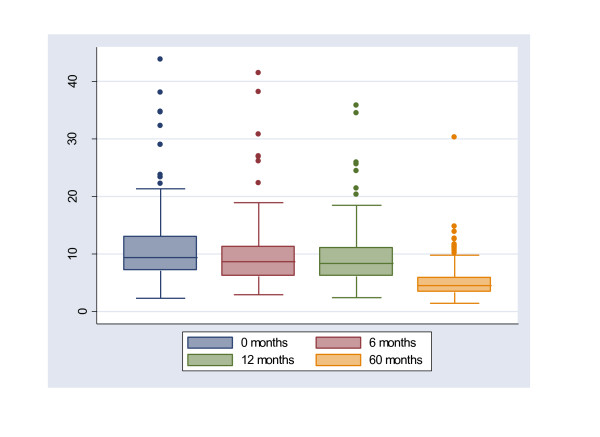
**Lead in blood concentrations of all children living in a smelter community studied during 60 months, in Torreon, Coahuila, Mexico**.

In order to determine differences between the children which continued with the follow-up study and those who did not, we compared media values of B-Pb, Hb and ZPP but found no significant differences in these parameters. At the onset of the study (0 time), the median value of B-Pb concentrations was slightly higher than 10.0 μg/dl (the safe accepted limit of the CDC [10]) and therefore, more than 50% of the children were over this value (Table 3). As time progressed, there was a clear decrease in B-Pb concentrations, which was statistically significant (correlation coefficient of - 0.3997, p < 0.05) (Figure 2). Anemia and increases of Zinc-Protoporphyrin were considered as effective biomarkers of lead toxicity. The risk for anemia (Hb < 12.4 g/100 mL of blood) was significantly higher at the start of the follow-up study, compared to the end of the study period (Table 3). There were no statistical significant changes in the timeline of Zinc-Protoporphyrin during the period of observation. In Table 1, we show the B-Pb concentrations in children by school. Schools 2 and 6 were those which presented higher B-Pb values at 0 months. On the other hand, schools 1 and 9, were located 3 km far away from the smelter. Schools 1 and 9 had similar values to those found by the control sites in our previous work [8].

**Table 3:** Timeline of B-Pb, Hb and ZZP in the group of lead exposed children during 60 months of follow-up.

	Time (months)			
Variable	0	6	12	60

B-Pb concentration (μg/dl) Median (range)	10.12 (1.9-43.8)	8.75 (1.85-41.45)	8.3 (2.4-35.8)	4.4 (1.3 - 30.3)

B-Pb ≥ 10 μg/dl (%)	50.84	39.46	37.42	5.6

Hemoglobin (g/dl)^b^	13.4 (10.0-16.5)	13.6 (10.2-16.6)	13.5 (11.2 - 15.9)	13.9 (10.7-15.9)

ZPP ^b ^(μmol ZP/mole Heme)	63.0 (10.0-224.0)	62.0 (25-197)	57 (34-180)	65 (16-248)

Hb < 12.4 g/dl (%)	10.02	7.32	7.23	4.32

Hb < 12.4 g/dl Risk ratio (95% IC)^ŧ^	2.41* (1.26 - 4.63)	1.76 (0.89 - 3.48)	1.74 (0.87 - 3.46)	1.0

ZPP ≥ 66 (μmol ZP/mole Heme) (%)	40.64	43.44	30.02	44.26

^ŧ ^As the last time (60 months) had the lower lead concentration in blood, this point was taken as the basal level of exposure.

^b ^Values are medians (range).

*. p < 0.05 two sided Fisher's exact test.

**Figure 3:**
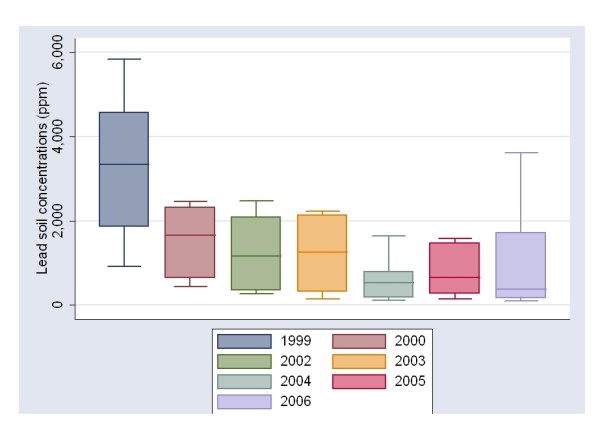
**Box plot representing the timeline of lead concentrations in soil of sites shown in Figure 1 in Torreon Coahuila, except sites 1, 8, and 9**. The first bar represents lead in soil concentration in 1999 as a basal time before environmental interventions began. The following bars represent lead in soil concentrations during the time the study was held, from 2002 to 2006. Superfund cleanup goal for lead is of 400 ppm [1].

We measured the concentrations of lead in soil in several neighborhoods, including those where the schools were located, in 1999, 2000, 2002, 2003, twice in 2004, twice in 2005 and lastly once in 2006. These results are evident in Figure 3, which shows a clear and significant decrease of lead concentrations in soil from 1999 to 2000, reaching a bottom level in 2004 and a slight tendency to increase in 2005. In table 4, we analyzed the relation between B-Pb with respect to Pb concentrations in soil after log transformation (2001, 2002 and 2006). The correlations among B-Pb were significantly dependent of Pb in soil at 0, 6, and 12 months of follow-up but the relationship was lost at 60 months, where B-Pb and Pb concentrations in soil were markedly lower than previous times. Lead in drinking water has always had very low concentrations in the Lagunera region, with values below 5 μg/L [8].

**Table 4:** Linear regression models of logarithm_10 _Pb in blood concentrations as dependent variables of logarithm_10 _Pb in soil concentrations, in the neighborhoods of the elementary schools were children lived at 0, 6, 12 and 60 months of follow-up in Torreon Coahuila.

Follow-up	n*	Coefficient (CI 95%)	Beta	P > | t |
0	196	0.095 (0.0494 - 0.1407)	0.283	0.000

6	196	0.074 (0.0279 - 0.1192)	0.222	0.002

12	196	0.130 (0.8336 - 0.1765)	0.367	0.000

60	196	- 0.009 (-0.6068 - 0.0433)	- 0.024	0.743

*.- There were 196 children who lived in the vicinity of the elementary school from where the soil samples were gathered.

CI. Confidence interval of regression coefficient at 95%.

## Discussion

This report shows the timeline of lead exposure in children resulting in lead environmental contamination in Torreon, Coahuila. The case of lead poisoning in Torreon captured international attention [15] and represented a challenge to developing countries, many of whom when looking for rapid industrialization, sometimes neglected the basic environmental control of hazardous metallurgic industries, resulting in severe metal pollution of environmental media, increasing the risk of toxic exposure of human populations to metals and other inorganic and organic pollutants [1]. Site studies showed that dust and soil were the main pathways to human exposure, particularly in children [8,15]. Until now, there had been no evidence indicating that drinking water had significant concentrations of lead and therefore, it did not represent a significant pathway for lead exposure [8]. Also, since 2000, lead concentrations in the air have reached consistently trimester averages < 1.5 μ/m^3^, which is the Mexican Standard [16].

When this follow-up study began in 2001, the median of B-Pb concentrations in 6 year old children was 10.12 μg/dl, this value was slightly higher than that obtained by the CDC in a cross sectional study [10] done the same year, where the geometric mean B-Pb of children was 8.3 μg/dl for the smelter area. These differences can be explained by: a) the cross-sectional design of the CDC study which covered the entire city, b) the age range in the CDC study was from 1 to 7 years, whereas in our study, the ages were more homogeneous, around 6-7 years old, and c) the analytical methods used by the CDC study determined B-Pb concentration by the portable anodic stripping voltammeter (LeadCare^® ^System, ESA Biosciences, Chelmsford MA), whereas we used AAS with a graphite furnace. Both methods have been very useful in determining B-Pb levels but there are discrepancies when both methods are applied. These differences be attributed to the fact that the Lead Care^® ^System increases the analytical variation in lower concentrations [17].

As expected, B-Pb levels were significantly related to lead in soil concentrations during the first twelve months but the relation was lost after five years. A possible explanation for the loss of relation is that after five years both lead in soil and lead in blood concentration decreases and loses the statistical association. Another rationale is statistical, since in the 2006 sampling, only 5.6% of children have B-Pb > 10.0 μg/dl (approximately 13 individuals) which is a very small number for an extreme segment of linear regression analyses. Yet one other contributive explanation is that 60 months of environmental measures and soil-dust cleaning activities could modify the geographical pattern of lead in soil distribution, however, this explanation does not agree with the general analyses of lead in soil results discovered by Villalobos-Jauregui et al. [14], where soil contamination is still strongly associated with the close proximity to the smelter complex.

The decrease in the exposure biomarker for lead (B-Pb) levels in the studied group could be explained by lower lead exposure due to improvements in environment quality. The evolution of this environmental case in Torreon Mexico is similar to that reported for Port Pirie in South Australia. As reported by Kranz et al. [18], in 1984 children had B-Pb concentrations with a geometric mean of 20 μg/dl (range 1 - 46 μg/dl). Since then, there has been a general downward trend to 9 μg/dl (1 - 4639 μg/dl) in 2002, which corresponded to environmental, nutritional, community and medical interventions in population. Aging can also decrease lead exposure by changes in children's habits, such as the normal abandon of mouth activities as children age [19]. Also, an important part of the decline in B-Pb can be explained by lead redistribution from blood to bone and other hard tissues, as a result of the usual toxicokinetics of lead [20]. It would be beneficial to study the lead in bone concentrations in this group when the children become adults.

In this work, we also studied the concentrations of hemoglobin and Zinc protoporphyrin as biomarkers of lead effect. Results showed that at the beginning of the study, when the children had the highest levels of lead exposure, they were more at risk for anemia, which is expressed as low hemoglobin levels (an adverse effect attributed to lead exposure) [10]. These results confirm the well described effects of lead on heme metabolism. Zinc protoporphyrin did not show significant changes during the study, possibly due to the fact that most of the children had B-Pb levels under the threshold for increased Zinc-protoporphyrin (91.6% of children had B-Pb levels below 20.0 μg/dl when the study began) [21].

## Conclusions

After an intensive intervention program done by authorities and the company, lead pollution in neighborhoods around the smelter complex known as Met-Mex Peñoles, in Torreon Mexico, has significantly decreased in magnitude, and therefore the program has been successful in the objective to decrease lead exposure in children, a fact that was reflected on the tested biomarkers of exposure in children. This could be explained by: a) environmental interventions by authorities and the smelter companies, b) normal changes in hygienic habits as children age and c) lead redistribution from blood to hard tissues. However, lead concentrations in soil still represent health risks for the human population. Although the blood lead values have decreased considerably in 2006, there are still 5.6% of children with B-Pb levels > 10 μg/dl. Therefore, lead exposure in the urban environment of Torreon needs more studies to address rational mitigation and remediation measures in order to decrease exposure to safe levels. Finally, follow-up studies should be encouraged to gain better scientific knowledge of metal exposure and the effect of mitigation and remediation measures in contaminated sites.

## List of abbreviations

B-Pb: Lead in blood expressed in μg/dl (conversion factor for SI units: 1 μg/dl = 0.04826 μmol/L); US CDC: United States Centers for Disease Control and Prevention.

## Competing interests

The authors declare that they have no competing interests.

## Authors' contributions

MRA participated as a project manager and coordinated the field work. FVP participated as responsible for Lead in soil determination and designed the soil sampling. JA participated as analytical responsible for lead in soil determination. JLR Participated in the Study Design. MEC participated as analytical responsible for lead in blood determination. GGGV participated in the general study design, and wrote the manuscripts. All authors read and approved the final manuscript.

## Endnotes

1 SSDC (Secretaría de Salud y Desarrollo Comunitario de Coahuila) (1999). Reportes técnicos a la mesa de seguimiento del problema con niños con altos niveles de plomo en sangre.

2 PROFEPA (Procuraduria Federal de Protección Ambiental) (1999). Evolución Reciente de la Contaminación Atmosférica Generada por Empresa Met-Mex Peñoles en Torreón.
